# Draft Genome Sequence of Calothrix Strain 336/3, a Novel H_2_-Producing Cyanobacterium Isolated from a Finnish Lake

**DOI:** 10.1128/genomeA.01474-14

**Published:** 2015-01-22

**Authors:** Janne Isojärvi, Sumathy Shunmugam, Kaarina Sivonen, Yagut Allahverdiyeva, Eva-Mari Aro, Natalia Battchikova

**Affiliations:** a Laboratory of Molecular Plant Biology, Department of Biochemistry, University of Turku, Turku, Finland; b Department of Food and Environmental Sciences, Division of Microbiology and Biotechnology, University of Helsinki, Helsinki, Finland

## Abstract

We announce the draft genome sequence of Calothrix strain 336/3, an N_2_-fixing heterocystous filamentous cyanobacterium isolated from a natural habitat. Calothrix 336/3 produces higher levels of hydrogen than Nostoc punctiforme PCC 73102 and Anabaena strain PCC 7120 and, therefore, is of interest for potential technological applications.

## GENOME ANNOUNCEMENT

To diversify a potential to exploit naturally occurring photosynthetic organisms for sustainable biofuel production, we investigated strains from the University of Helsinki Culture Collection (UHCC) for their capability to produce H_2_ (1). The preliminary screening process involved 400 different cyanobacterial species utilizing four different conditions, including aerobic, anaerobic, light, and dark conditions. The promising strains were then compared against the H_2_ photoproduction capacities of reference strains Nostoc  punctiforme PCC 73102 and Anabaena PCC 7120 cyanobacteria. After optimization of the production system and exclusion of strains that had a toxic nature or have been too light sensitive, two strains, Calothrix 336/3 and Calothrix XPORK 5E, have been found to be the best H_2_-producing cyanobacteria among investigated species (1, 2).

Calothrix 336/3 is an N_2_-fixing heterocystous filamentous cyanobacterium isolated from the Enäjärvi lake, Laukilanlahti, Finland (3). DNA preparations were obtained as described by Neilan et al. (4) and sent for commercial sequencing at the Beijing Genome Institute (BGI), where two Calothrix 336/3 DNA libraries containing short ~500-bp inserts and mate-pair ~2,000-bp inserts were constructed. Sequencing was performed on the HiSeq 2000 Illumina platform. A total of 13,333,340 raw paired-end reads were produced, resulting in ~100-fold genome coverage. The reads were assembled with SOAPdenovo (5) into 43 scaffolds larger than 100 bp. The following GC-content analysis showed the presence of the two scaffold groups. The GC content of 41% to 50% was determined for 27 scaffolds, while 16 scaffolds had a GC content of 30% to 34%. The taxonomic analysis with MEGAN v. 5.5.4 (6) revealed that the first group of 27 scaffolds contained proteins that were found in other cyanobacterial species, while the second group belonged to an unknown *Bacteroides*-like genus, which resulted, most probably, from strain contamination. The latter group of 16 contaminated scaffolds was discarded from further analysis. The 27 scaffolds with the higher GC content were combined by PCR using synthetic primers selected from regions near scaffold ends, resulting in 4 scaffolds, with a total of 6,419,212 bp.

The draft genome was automatically annotated with the NCBI Prokaryotic Annotation Pipeline (PGAP); (http://www.ncbi.nlm.nih.gov/genomes/static/Pipeline.html), using the Rapid Annotations based on Subsystem Technology (RAST) server (7, 8) and the DOE-JGI Microbial Annotation Pipeline (9). The resulting Calothrix 336/3 genome comprises 4,946 genes with 4,834 coding sequences, 42 pseudogenes, 6 ribosomal RNAs, and 63 transfer RNAs. The overall GC content percentage is 41.41%. Based on 16S rRNA gene sequence similarity, Calothrix 336/3 is 93.73%, 92.45%, and 91.98% identical to the Calothrix strains PCC 7507 (CP003943), PCC 6303 (CO003610), and PCC 7103 (ALVJ00000000), respectively.

Genome annotation revealed that the Calothrix 336/3 genome contains *nif* (*nifHDK1*) and *hup* (*hupLS*) operons encoding nitrogenase and uptake hydrogenase enzymes but lacks *hoxEFUYH* genes encoding bidirectional hydrogenase and sets of *nifHDK2* and *vnfDGK* genes encoding alternative nitrogenases, in line with the results obtained by Leino et al. with enzyme activity and Southern hybridization analyses (2). The strain demonstrated a high stability and prolonged H_2_ photoproduction capacity after immobilization in thin alginate films (10, 11). Investigation of the Calothrix 336/3 genome opens new opportunities for potential technological applications in the development of biohydrogen production.

### Nucleotide sequence accession number.

The draft genome sequence of Calothrix strain 336/3 has been deposited at GenBank under the accession number JPKF00000000.

## References

[1] Allahverdiyeva Y, Leino H, Saari L, Fewer DP, Shunmugam S, Sivonen K, Aro E 2010 Screening for biohydrogen production by cyanobacteria isolated from the Baltic Sea and Finnish lakes. Int J Hydrogen Energ 35:1117–1127. doi:10.1016/j.ijhydene.2009.12.030.

[2] Leino H, Shunmugam S, Isojärvi J, Oliveira P, Mulo P, Saari L, Battchikova N, Sivonen K, Lindblad P, Aro E, Allahverdiyeva Y 2014 Characterization of ten H_2_ producing cyanobacteria isolated from the Baltic Sea and Finnish lakes. Int J Hydrogen Energ 39:8983–8991. doi:10.1016/j.ijhydene.2014.03.171.

[3] Sihvonen LM, Lyra C, Fewer DP, Rajaniemi-Wacklin P, Lehtimäki JM, Wahlsten M, Sivonen K 2007 Strains of the cyanobacterial genera *Calothrix* and *Rivularia* isolated from the Baltic Sea display cryptic diversity and are distantly related to *Gloeotrichia* and *Tolypothrix*. FEMS Microbiol Ecol 61:74–84. 17466025 10.1111/j.1574-6941.2007.00321.x

[4] Neilan BA, Jacobs D, Goodman AE 1995 Genetic diversity and phylogeny of toxic cyanobacteria determined by DNA polymorphisms within the phycocyanin locus. Appl Environ Microbiol 61:3875–3883. 8526499 10.1128/aem.61.11.3875-3883.1995 PMC167692

[5] Luo R, Liu B, Xie Y, Li Z, Huang W, Yuan J, He G, Chen Y, Pan Q, Liu Y, Tang J, Wu G, Zhang H, Shi Y, Liu Y, Yu C, Wang B, Lu Y, Han C, Cheung DW, Yiu SM, Peng S, Xiaoqian Z, Liu G, Liao X, Li Y, Yang H, Wang J, Lam TW, Wang J 2012 SOAPdenovo2: an empirically improved memory-efficient short-read *de novo* assembler. GigaScience 1:18. doi:10.1186/2047-217X-1-18. 23587118 PMC3626529

[6] Huson DH, Mitra S, Ruscheweyh HJ, Weber N, Schuster SC 2011 Integrative analysis of environmental sequences using MEGAN4. Genome Res 21:1552–1560. doi:10.1101/gr.120618.111. 21690186 PMC3166839

[7] Aziz RK, Bartels D, Best AA, DeJongh M, Disz T, Edwards RA, Formsma K, Gerdes S, Glass EM, Kubal M, Meyer F, Olsen GJ, Olson R, Osterman AL, Overbeek RA, McNeil LK, Paarmann D, Paczian T, Parrello B, Pusch GD, Reich C, Stevens R, Vassieva O, Vonstein V, Wilke A, Zagnitko O 2008 The RAST server: Rapid Annotations using Subsystems Technology. BMC Genomics 9:75-2164-9-75. doi:10.1186/1471-2164-9-75. 18261238 PMC2265698

[8] Overbeek R, Olson R, Pusch GD, Olsen GJ, Davis JJ, Disz T, Edwards RA, Gerdes S, Parrello B, Shukla M, Vonstein V, Wattam AR, Xia F, Stevens R 2014 The SEED and the rapid annotation of microbial genomes using subsystems technology (RAST). Nucleic Acids Res 42:206. doi:10.1093/nar/gkt1226. PMC3965101 24293654

[9] Mavromatis K, Ivanova NN, Chen IM, Szeto E, Markowitz VM, Kyrpides NC 2009 Standard Operating Procedure for the Annotations of Microbial Genomes by the Production Genomic Facility of the DOE JGI. Stand Genomic Sci 1:63–67. doi:10.4056/sigs.632. 21304638 PMC3035208

[10] Leino H, Kosourov SN, Saari L, Sivonen K, Tsygankov AA, Aro E, Allahverdiyeva Y 2012 Extended H_2_ photoproduction by N_2_-fixing cyanobacteria immobilized in thin alginate films. Int J Hydrogen Energ 37:151–161. doi:10.1016/j.ijhydene.2011.09.088.

[11] Kosourov S, Leino H, Murukesan G, Lynch F, Sivonen K, Tsygankov AA, Aro EM, Allahverdiyeva Y 2014 Hydrogen photoproduction by immobilized N_2_-fixing cyanobacteria: understanding the role of the uptake hydrogenase in the long-term process. Appl Environ Microbiol 80:5807–5817. doi:10.1128/AEM.01776-14. 25015894 PMC4178605

